# Feeding Animals1Read at the annual meeting of the Pennsylvania State Veterinary Medical Association, March, 1898.

**Published:** 1898-04

**Authors:** J. Curtis Michener

**Affiliations:** Colmar, Pa.


### FEEDING ANIMALS.[1]

By J. Curtis Michener, V.S.,
COLMAR, PA.

This subject was selected, not that feeding animals is any special part of the veterinarian's duty, but quite as important for him to understand as the stockman whom he serves. More than this, his client has a right to expect of him sound counsel upon any of the manifold problems that arise in the healthful and economical feeding of all animals and poultry. His practised eye should be quick to detect any deviation from the perfect thrift that marks the animal when at its best, for the purpose for which it is being fed, and be able to prescribe the necessary diet to correct defective conditions, instead of giving condition-powders.

However, they act admirably when given together. The basis of all intelligent and successful feeding is in the recognition of the underlying fact that the various feeds are composed of the same elements as the bodies they nourish. In other words, vegetation incorporates from soil and air the materials that the animal body is about to appropriate. But the proportions are seldom right. The water, ash, protein, fat, and carbohydrates of the various forage-plants and cereals are in widely varying proportions and degrees of digestibility ; so that it is possible to starve an animal while giving it all it is able to eat, to greatly curtail the production of milk by a badly-balanced ration, or to so diminish force as to render an animal worthless for work. To feed for bare maintenance is one thing ; for rapid growth and full development, a better thing ; to feed a milk-cow at a loss, an easy thing ; to feed a

[1] Read at the annual meeting of the Pennsylvania State Veterinary Medical Association, March, 1898.

horse up to his full capacity for work, a grand thing. While pedigree is important, skill in feeding makes the successful breeder.

We have different kinds of feeding. Scientific feeding: the right materials in exact proportions for desired results; good feeding, derived from the experience of one's self, and teaching of others; hap-hazard feeding, and ignorant, careless, ruinous, criminal feeding. But our live-stock interests, the greatest of any people upon the globe, demand that we understand and practise the business for the best results. Science is the lever; experience the hand that applies it; love and admiration for our animals, the inspiration, and profit accruing, the consummation devoutly wished for.

The science of feeding is exact so far as determining the relative proportions of the digestible protein to the carbohydrates and fat, for different purposes, under the same conditions. Animals are kept under such widely different circumstances as to shelter, ventilation, exercise, and work, and have formed different habits from influence of environment, making it hard to lay down inflexible rules. The analysis of the different foods at hand, and a careful study of the condition, purpose, and characteristics of the animal enable the skilful feeder to acquire exactness and proficiency.

I will enumerate the essential principles of the art of feeding, in the order of their importance, but must omit detail from want of time. As before intimated, feeding is reduced to an exact science; and knowledge of the composition of animal bodies, and of the various foods that are to sustain, make growth, produce milk or wool, repair waste, perform work and lay on fat must be familiar, and by the use of figures which will not lie, the problem is solved.

The materials must be so selected and combined as to constitute the balanced ration for the purpose. Animals are soon fed and bred into a fat-forming or beef-habit which destroys their adaptability for the dairy or the racetrack; hens fed exclusively upon fattening foods cease laying; the hogs of our own section fail to fill the market demand—too much corn-fed, too little exercise, too much lard. The chemical constitution of the feed is the chief factor in giving firmness and hardness of bone and muscle-tone and action. Too little attention is paid to the amount of water in the feed. We see animals being nearly physicked to death upon succulent foods, and others badly impaired by constipation caused by hard, dry food. The right condition is maintained by proper combinations of feed. Stock need roots, silage, wet feed, or mash. The amount of needed water varies with the purpose of the animal, and cannot be supplied by drink alone. The time of cutting and the perfection of the drying or curing process go far in determining the palatableness, the digestibility, and the danger of undergoing fermentation in the digestive tract. Kiln-dried, finely-ground cereals are the safest and most healthful, mixed with silage or made into a mash with cut-fodder or hay. From contact, I know the average farmer and feeder is not educated up to these points. Can he obtain the needed knowledge from his veterinarian? The problem is sometimes difficult because of the limited material at hand, and the price of such stuff as would balance up the ration being so high as to make its use unprofitable. Then the question is, What is best under the circumstances? Having determined this important matter, the quantity to be fed comes next. Medium or average quantities for different ages, weights, and purposes should be known; but individual capacity, natural and acquired, must be found out. Only liberal feeding is profitable; under- and overfeeding are mistakes. The varying values of feed in the manure must not be lost sight of when among the farmers. It is nice to be a skilful mechanic and construct useful things, or to understand the running of machinery; but such are not to be compared to the man who can grow and fatten animals just right, or to him who can run a herd of dairy-cows so as to get all from them that is to be had, and avoid indigestion, garget, and concomitant dangers and losses. It is done by regularity in watering and feeding, avoiding exposure or sudden changes in diet; gradually increasing the feed upon new animals until their capacity is determined, then keeping a sharp watch for the first indication of surfeit, and withholding until the keen appetite returns. The condition of the atmosphere, the temperature, the amount of fresh air entering the stable, and the exercise, all influence the appetite and digestion, and are taken into account by the practical feeder. Some feed may analyze well, but not be relished by stock, as individual animals have their likes and dislikes the same as persons, which opens a field for observation and tact. The addition of salt makes feed more palatable and digestible. A milk-cow should consume two ounces per day mixed through the feed; a work-horse one ounce. Cheap sugar and molases can be profitably used; also various condiments. We should not allow the patent-feed and medicine-man to monopolize these things.

---

Michigan's State Veterinarian is kept busy among reported outbreaks of disease in cattle and swine.

